# Influence of Cement Replacement with Fly Ash and Ground Sand with Different Fineness on Alkali-Silica Reaction of Mortar

**DOI:** 10.3390/ma14061528

**Published:** 2021-03-20

**Authors:** Suwat Ramjan, Weerachart Tangchirapat, Chai Jaturapitakkul, Cheah Chee Ban, Peerapong Jitsangiam, Teewara Suwan

**Affiliations:** 1 Building Control Bureau, Department of Public Works and Town & Country Planning, Bangkok 10140, Thailand; tience14@hotmail.com; 2 Construction Innovations and Future Infrastructure Research Center (CIFIR), Department of Civil Engineering, Faculty of Engineering, King Mongkut’s University of Technology Thonburi, Bangkok 10140, Thailand; Chai.jat@kmutt.ac.th; 3 School of Housing, Building and Planning, Universiti Sains Malaysia, Penang 11800, Malaysia; cheahcheeban@usm.my; 4 Center of Excellence in Natural Disaster Management, Department of Civil Engineering, Faculty of Engineering, Chiang Mai University, Chiang Mai 50200, Thailand; peerapong@eng.cmu.ac.th (P.J.); teewara.s@cmu.ac.th (T.S.)

**Keywords:** alkali-silica reaction, blended cement, filler, cement-based materials, fly ash, mortar

## Abstract

The alkali-silica reaction (ASR) is an important consideration in ensuring the long-term durability of concrete materials, especially for those containing reactive aggregates. Although fly ash (FA) has proven to be useful in preventing ASR expansion, the filler effect and the effect of FA fineness on ASR expansion are not well defined in the present literature. Hence, this study aimed to examine the effects of the filler and fineness of FA on ASR mortar expansion. FAs with two different finenesses were used to substitute ordinary Portland cement (OPC) at 20% by weight of binder. River sand (RS) with the same fineness as the FA was also used to replace OPC at the same rate as FA. The replacement of OPC with RS (an inert material) was carried out to observe the filler effect of FA on ASR. The results showed that FA and RS provided lower ASR expansions compared with the control mortar. Fine and coarse fly ashes in this study had almost the same effectiveness in mitigating the ASR expansion of the mortars. For the filler effect, smaller particles of RS had more influence on the ASR reduction than RS with coarser particles. A significant mitigation of the ASR expansion was obtained by decreasing the OPC content in the mortar mixture through its partial substitution with FA and RS.

## 1. Introduction

Fly ash (FA) is one of main by-products from coal combustion in thermal power plants. Currently, the Mae Moh power plant in Thailand produces about 2.1 million tons of fly ash, increasing annually. Fly ash (FA) is also a well-known mineral admixture and has been widely accepted for use as a pozzolanic material to partially replace ordinary Portland cement (OPC) in concrete. The use of fly ash as a pozzolanic material is constantly increasing because it improves the properties of mortar and concrete, namely the workability, durability, and strength in the long term. Generally, fly ash from pulverized combustion has a spherical particle shape and high fineness. However, some fly ashes also contain irregular or angular particles. The size and shape of fly ash particles vary depending on the source and combustion conditions. The chemical composition of fly ash also depends on the characteristics of the coal and burning condition. FA has been studied for a long time and has been continuously developed to improve many of the properties of concrete. In 2010, Sharma et al. [1] studied the influence of FA characteristics from 14 thermal power plants and found that calcium content and particle size are the main parameters affecting the compressive strength of concrete. Compressive strength is an important factor to consider when selecting concrete mix proportions, and the use of FA can provide compressive strengths as high as OPC concrete [2,3,4,5]. Moreover, replacing OPC with FA in the concrete mixture can produce high strength concretes, with compressive strengths of 77.3 to 82.5 MPa at an age of 28 days [6]. Once FA was accepted as a way to improve the compressive strength of concrete, many researchers studied FA for improving other properties of concrete, such as workability and durability. The use of FA can significantly improve the elementary properties of paste and mortar, such as normal consistency, setting time, and water requirements [7].

In terms of durability, the replacement of 20 to 50% of OPC with FA (by weight of binder) has been shown to produce concrete with lower water permeability than concrete without FA. In addition, similar FA contents have been used to produce concrete with a higher resistance to sodium and magnesium sulfates than OPC concrete [8]. FA is a popular pozzolan for preventing chloride penetration into concrete in marine environments [5]. The use of FA also contributes to a lower chloride penetration depth of recycled aggregate concrete than that of OPC concrete, by approximately 2–2.5 times for recycled aggregate concrete without FA [8]. Additionally, FA has been used to improve both the fresh and mechanical properties of concrete containing recycled aggregate [2]. FA has also been used as a major cementing material to produce calcium carbide residue-FA concrete with zero OPC [9] and alkali-activated materials [10].

Turk et al. [11] confirmed that FA is a good pozzolan for use in controlling alkali-silica reactions (ASRs). It had been established that the use of low-calcium Class F FA at 20% blended with limestone powder can mitigate ASR expansion. Saha et al. [12] stated that the use of Class F FA can reduce ASR expansion more significantly than Class C FA. However, the findings of Moser et al. [13] showed that the use of FA does not always help to reduce the ASR expansion of mortars with very highly reactive aggregates. In their study, it was reported that the control mortar had an ASR expansion of 0.70% at 14 days. Meanwhile, replacing 20% of the OPC with Class C FA failed to reduce the ASR to below 0.20% at 14 days.

Although FA has been proven by many researchers to be a useful pozzolanic material for preventing ASR expansion, the primary reduction of the expansion may be due to other factors, such as the filler effect or the reduction of the OPC content in the mixture. Additionally, the filler effect and the effects of FA fineness on ASR expansion have not been well defined in the present literature. Therefore, this investigation was aimed at examining the influence of the replacement of OPC with FA with different levels of fineness on the ASR expansion of mortar. Two fineness levels of FA were also considered in order to observe the effect of fineness on ASR expansion. Ground river sand (RS) with the same fineness as FA was used to replace OPC at the same rates of FA to study the filler effects of FA and RS on the ASR expansion of mortar.

## 2. Research Significance

Alkali-silica reaction (ASR) is a dangerous phenomenon in concrete structures, because ASR builds internal pressure causing the concrete to crack. After that, the concrete structure losses its compressive strength and durability, making it easy for moisture and other chemical solutions to migrate into the internal concrete, and leading to the corrosion of steel in reinforced concrete structures. Although, the replacement of OPC by fly ash can reduce the expansion due to ASR, other factors are still not clear, such as the filler effect and the effects of the fineness of the materials. Therefore, the knowledge obtained from this study will help the civil engineer to understand the factors affecting the ASR expansion of mortar, and lead to applying this knowledge in concrete structures in order to obtain the safety and sustainability of structures.

## 3. Experiment

### 3.1. Materials

A low alkali cement obtained from a cement manufacturer in Thailand, which was classified as type I according to ASTM C150 [14], was used to cast all mortars. Fly ash (FA) was collected from the Mae Moh electricity generating plant, Thailand, where lignite coal is burned using a pulverized burning system. The FA was obtained directly from the plant and was used as received. The fineness of the FA was measured by weighing particles of FA retained on a No. 325 sieve [15]. The original FA had 32.8% of its particles retained on the No. 325 sieve, which does not exceed the limit defined by ASTM C618 [16] of 34%. This FA was called “33FA”. The FA was then ground by a ball mill to have 5.0% of its particles by weight retained on a No. 325 sieve (5FA). River sand was used as an inert material in order to study the filler effect on the ASR expansion of the mortar. River sand used in this study was collected from the central part of Thailand. To examine the effect of the fineness of FA on ASR, the RS was also ground to have the same fineness as the FA.

**Figure 1:**
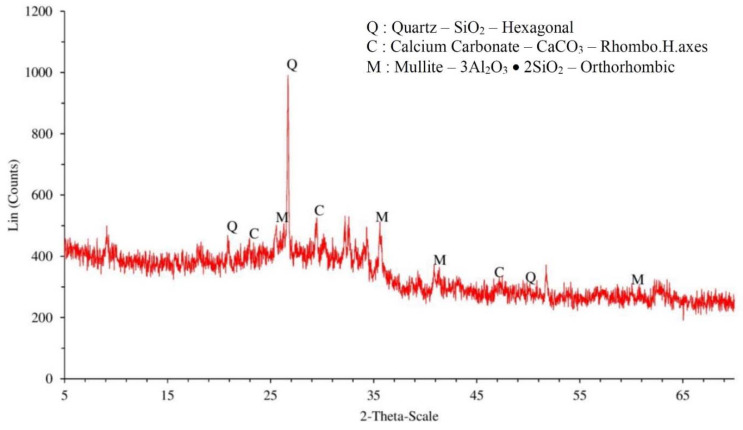
The qualitative results (XRD) of 5FA.

**Figure 2:**
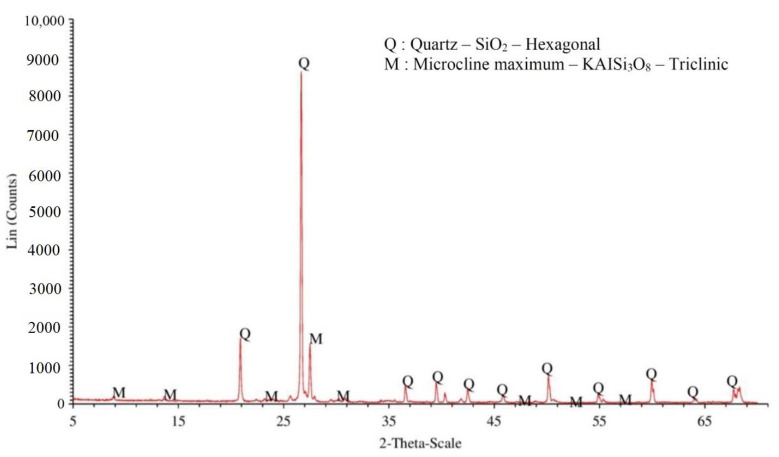
The qualitative results (XRD) of 5RS.

After completing the grinding processes, the crystallinity of the 5FA and 5RS were analyzed by X-ray diffraction (XRD), and the results are presented in Figure 1 and Figure 2, respectively. The 5FA had crystals of quartz (SiO_2_), calcium carbonate (CaCO_3_), and mullite (3Al_2_O_3_·2SiO_2_), while the 5RS had mainly crystals of quartz. However, the quartz in RS does not react with Ca(OH)_2_ and was treated as an inert material, since the area under the XRD graph was very low. The XRD results of 33FA and 33RS were not analyzed because the results of 33FA and 33RS were assumed to be similar to those of 5FA and 5RS, respectively. Chindaprasirt et al. [17] and Kroehong et al. [18] proved that fly ash and palm oil ash (with the same source) with different finenesses had only slight differences in their XRD results.

Many prior studies have established that the fineness of pozzolanic materials has little influence on their chemical compositions [19]. The chemical properties of 5FA met the requirements of Class C FA according to ASTM C618 [16], which requires that the summation of SiO_2_ + Al_2_O_3_ + Fe_2_O_3_ is more than 50%, SO_3_ is less than 5%, and loss on ignition is less than 6%, while the alkali equivalent (Na_2_O_eq_) is 2.8% by weight. The major oxide of 5RS is SiO_2_, which makes up 92.5% of its content, and the rest are other oxides. The chemical compositions of the materials are given in Table 1, and Table 2 shows the physical properties and strength activity index of materials.

**Figure 3:**
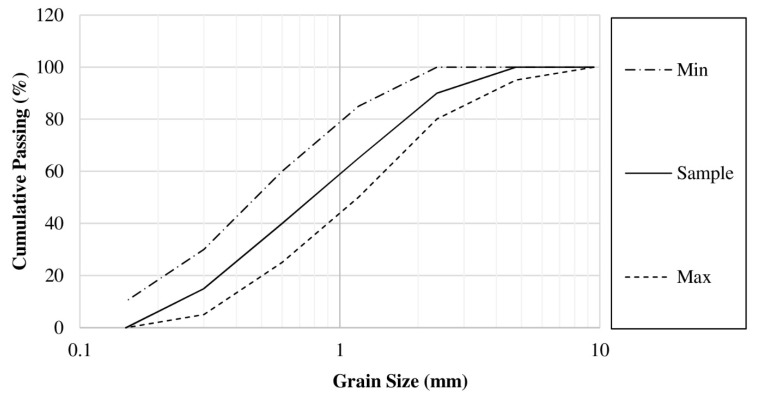
Particle size distributions of the reactive fine aggregate used in this study.

For mortars containing an ASR reactive aggregate, the coarse reactive aggregate was ground and sieved until the gradation met the requirements of ASTM C1567 [20] for ASR investigation. The fine reactive aggregate had a specific gravity (saturated surface dry, SSD) and fineness modulus of 2.63 and 2.90, respectively. The gradation of the fine reactive aggregates in this study was compared with the required gradation of fine aggregates specified in ASTM C1260 [21], as shown in Figure 3.

### 3.2. ASR Testing Procedure

The ASR of the mortar was investigated by an accelerated method, and according to ASTM C1567 [20]. For the first step, a binder to fine aggregate ratio of 2.25 was kept, while a water to binder (W/B) ratio of 0.47 was used to cast mortar bars. It is noted that the W/B ratio of the mortar bars was calculated with the effective water content, which already compensates for the water absorption by the fine reactive aggregate. For the binder in the mortar bar mixtures, FA and RS were used to partially replace OPC at 20% by weight of binder. The mix proportions of the mortar bars, including their flows, are presented in Table 3.

Next, two stainless steel gauge studs were cast-in at both ends of the mortar bar mold for use as an indicator in the measuring process. A mortar mixture was filled and compacted in three molds with dimensions of 25 × 25 × 285 mm^3^. Mortar bars were de-molded after 24 h of casting and were then cured in tap water at a temperature of 80 °C. The initial length of the mortar was measured 24 h after completion of the previous process. Next, the mortar bars were immersed in a 1 N sodium hydroxide (NaOH) solution at the temperature of 80 °C. The length of mortar bars was read for 28 days and the reading at each time was targeted to be the nearest 0.001% of the nominal gauge length. The reported ASR expansion was presented as the percent change in length, which was obtained from the average of three mortar specimens. The acceptable range of test results between samples at the same testing age and mix proportion was 8.3% of the average as specified by ASTM C1567 [20].

The effectiveness of using fly ash or ground river sand in controlling the ASR expansion of mortars was determined according to Equation (1), given by ASTM C441 [22] as follows:

(1) $$R_{e}=\frac{E_{ct}-E_{t}}{E_{ct}}\times 100$$

where *R_e_* is the reduction of the expansion of mortar (%), *E_t_* is the average length of mortar bars containing fly ash or ground river sand (%), and *E_ct_* is the average length of the control mortar bars (%).

## 4. Results and Discussion

### 4.1. ASR Expansion of Mortars Containing FA

**Figure 4:**
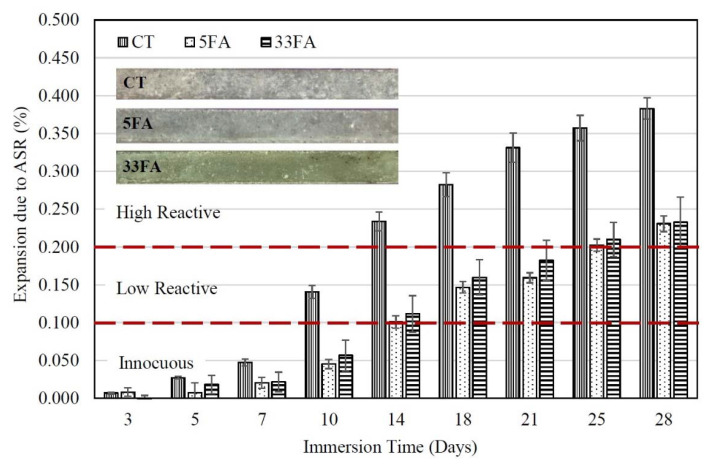
Relationship between the ASR expansion of 5FA and 33FA mortars and immersion time.

As shown in Figure 4, the control mortar (CT), in which the crushed reactive coarse aggregate was used as a fine reactive aggregate, had an ASR expansion of 0.234 ± 0.013% at 14 days, exceeding 0.20% and suggesting that the fine aggregate used should be classified as a highly reactive aggregate according to ASTM C1567 [20]. In addition, this highly reactive aggregate had a high ASR mortar expansion similar to trachyte, trachyandesite, rhyolite, and glass aggregates [23,24]. Various mechanisms for the occurrence of ASR in mortar or concrete without pozzolan have been suggested by studies. Several researchers [25,26] have found that increasing the amount of Ca(OH)_2_ promotes a high yield of ASR product. Additionally, Hou et al. [27] demonstrated a direct relationship between the addition of OPC and the development of ASR gels. The formation of ASR expansion products was found in the CT mortar because the NaOH solution provided a high alkalinity level, while the primary calcium source was released by OPC during the hydration process [28]. However, the reduction of the OPC content in the mixture through the partial replacement of OPC by added minerals or pozzolanic materials could reduce the amount of Ca(OH)_2_, thereby reducing the degree of ASR expansion.

The ASR expansions of mortars containing 5FA and 33FA is presented in Figure 4. Mortars containing either 5FA or 33FA at 20% had ASR expansions that were lower than the CT mortar. The 5FA and 33FA mortars had expansions of 0.101 ± 0.008% and 0.112 ± 0.010% at 14 days, which increased to 0.231 ± 0.010% and 0.233 ± 0.020% at 28 days, respectively. The partial substitution of OPC with FA or other pozzolans in the mortar mixture was shown to reduce the ASR expansion, which is consistent with the findings of a number of prior studies [26,29,30]. In this study, the degree of fineness of the FA used as a replacement for OPC had a marginal influence on the degree of ASR expansion of the mortars. This is consistent with the reported findings of Aydin et al. [31], who found that changing between the original and ground FA from the same source had no significant influence on the degree of ASR expansion. For example, mortars incorporating 5FA and 33FA had expansions at 14 days of 0.101 ± 0.008 and 0.112 ± 0.010%, respectively. While the results of Aydin et al. [31] showed that ASR expansions at 14 days of mortars containing original and ground fly ash were approximately 0.095 and 0.085%, respectively. It was clearly seen that a reduction in the fineness of fly ash could decrease the expansion of mortar due to an ASR of not more than 5%.

A mechanism for the reduction of the ASR expansion due the addition of pozzolan was described by Topçu et al. [30], who reasoned that the pozzolanic reaction between FA and Ca(OH)_2_ (from the cement hydration reaction) leads to a decreased pH in the pore solution. Lindgård et al. [32] stated that supplementary cementitious materials (SCMs) made from low CaO and high SiO_2_ pozzolans are the most critical factors in reducing the alkalinity of pore solutions. The corresponding reduction in the concentration of hydroxyl ions in the pore solutions could prevent ASR expansion or reduce its degree. Conversely, the FA used in the current study had a high amount of CaO (25.2 wt.%) and a low amount of SiO_2_ (33.2 wt.%), but it also reduced the expansions of the mortars. Topçu et al. [30] explained that the pozzolanic reaction of FA could reduce the reactivity between the alkali and silica due to the formation of a dense paste structure. This also results in the subsequent reduction in the amount of water or solution which is able to penetrate the paste matrix. A higher proportion of OPC replacement by FA or other pozzolans has been shown to establish a lower Ca(OH)_2_ content in the hardened paste. Moreover, it also decreases the availability of alkali reactants, due to the partial consumption of Ca(OH)_2_ by pozzolanic reaction [13,33,34,35]. In addition, Hong and Glasser [36] suggested that the Al_2_O_3_ content might also have an important influence on the alkali capacity of an SCM binder.

The reduction of ASR expansion in mortar or concrete containing FA is attributed to the reduction of alkali in the binder. However, the main factor that limits the ASR expansion is the lower amount of OPC in the mixture, achieved by using FA as a partial substitute for OPC. To verify this claim, RS (inert material) was used to replace OPC instead of FA, to demonstrate the effect of the cement content on ASR expansion.

### 4.2. ASR Expansion of Mortars Containing RS

**Figure 5:**
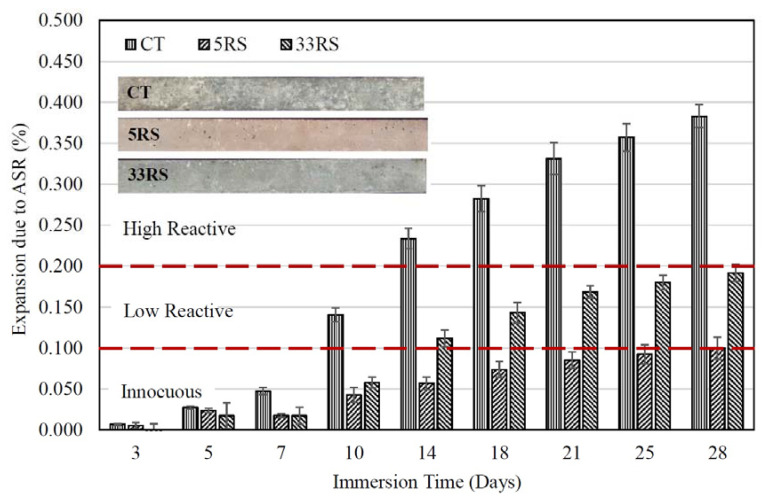
Relationship between the ASR expansion of 5RS and 33RS mortars and immersion time.

Figure 5 shows the relationship between the ASR expansions of RS mortars and immersion time. It is interesting to note that the replacement of OPC by 5RS and 33RS in mortars resulted in lower ASR expansions as compared to the CT mortar. The 5RS mortar had an ASR expansion below 0.10% at 14 days, indicating that a 20% 5RS content could reduce the degree of ASR expansion of mortar from a highly reactive level to an innocuous level (less than 0.10%). However, it should be noted that RS is ground river sand, which is an inert material due its high crystallinity, as shown in Figure 2. Thus, the mitigation of ASR expansion was a result of the decrease of the OPC content in the binder matrix.

The replacement of OPC with 5RS (high fineness) reduced the ASR expansion of the mortar more than replacement with 33RS (low fineness), because the 5RS particles could better fill voids and resist the penetration of the 1 N NaOH solution into the mortar. For instance, the 5RS mortar had an ASR expansion of 0.061 ± 0.003% at 14 days, while that of the 33RS mortar was 0.112 ± 0.009%. The fineness of RS is one significant factor that influences the reduction of ASR in mortar. Finer RS also provided a higher compressive strength to the mortar than RS with lower fineness, largely due to the filler effect [37].

Although the replacement of OPC by RS can reduce ASR expansion, it can also result in a lower compressive strength of mortar [37,38,39]. Thus, the utilization of RS in controlling the ASR expansion must also take into account the required compressive strength of mortar.

### 4.3. ASR Expansion of Mortars Containing FA and RS

**Figure 6:**
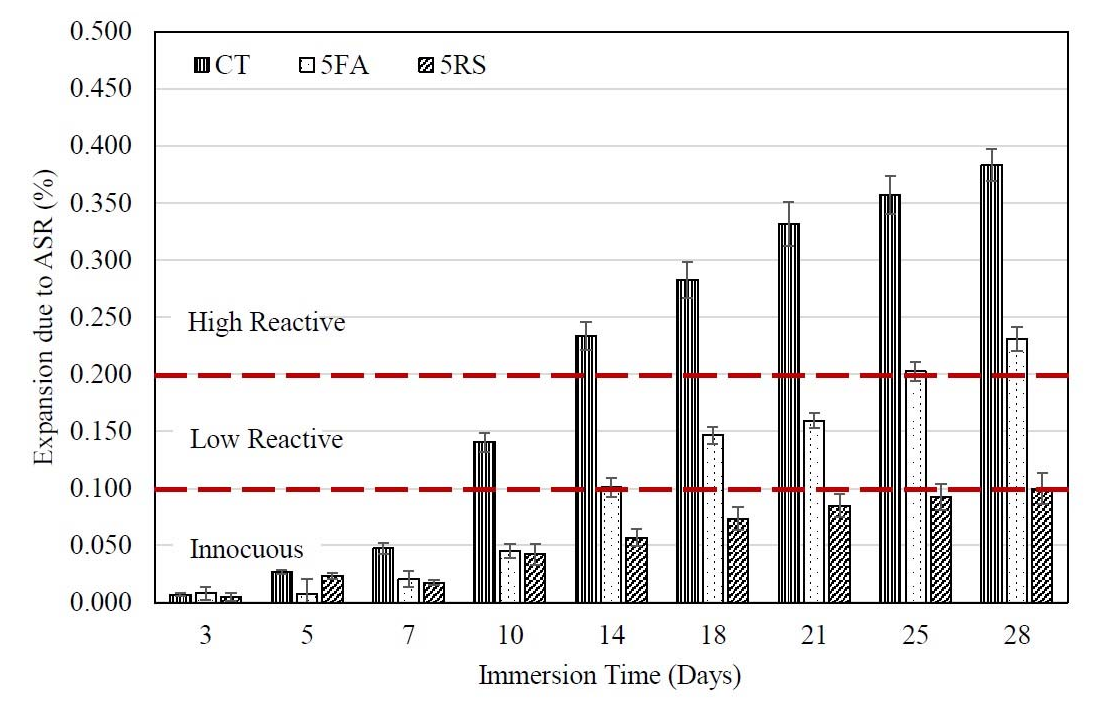
Relationship between the ASR expansion of 5FA and 5RS mortars and immersion time.

**Figure 7:**
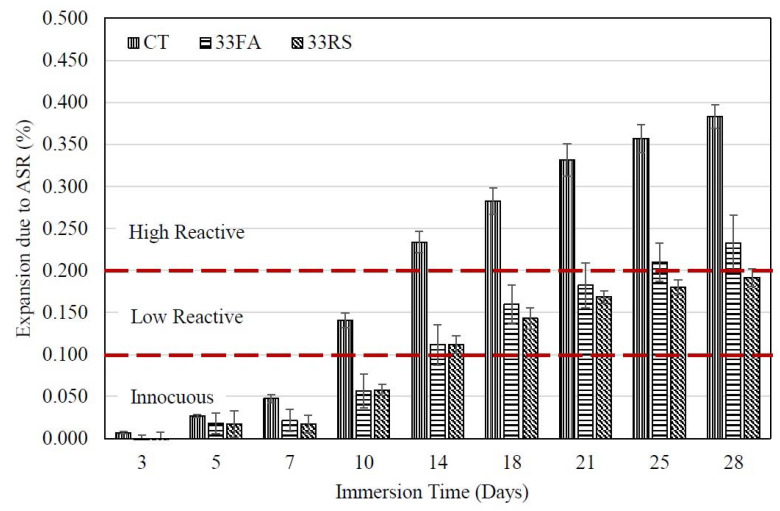
Relationship between the ASR expansion of 33FA and 33RS mortars and immersion time.

Figure 6 shows the relationship between the ASR expansion of the 5FA and 5RS mortars and immersion time. The expansion of the 5FA mortar was higher than for the 5RS mortar at 28 days. However, the ASR expansion of the 5FA mortar was similar to that of the 5RS mortar at periods before 10 days. This is largely due to the slow pozzolanic reaction of FA at early ages. After 10 days, the expansion of the 5FA mortar continuously increased until it reached 0.231 ± 0.010% at 28 days, while the 5RS mortar had an ASR expansion of 0.093 ± 0.007% at 28 days. The increase in the ASR expansion of the 5FA mortar was more than for the 5RS mortar because the CaO in 5FA increases the alkalinity of the mixture. A similar behavior could be observed in the 33FA and 33RS mortars (see Figure 7), in which the non-reactive period of the 33FA mortar was marginally longer than that of the 5FA mortar (before 14 days). On the other hand, the difference in the ASR expansion between the 33FA and 33RS mortars was lower than the difference between the 5FA and 5RS mortars because the coarser CaO in 33FA reacted more slowly and less completely than the finer CaO in 5FA.

### 4.4. Filler Effect of Different Finenesses of RS on the ASR Expansion of Mortar

**Figure 8:**
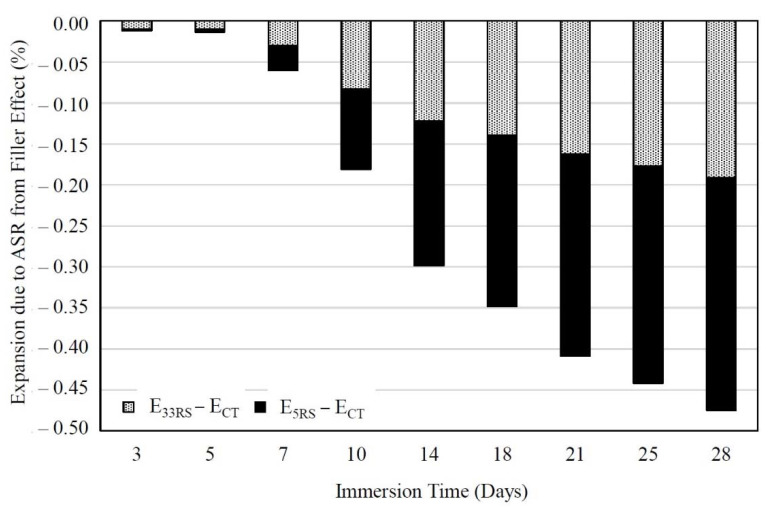
Filler effect of 5RS and 33RS particles on the ASR expansion of mortars.

Figure 8 shows the relationship between the ASR expansions of RS mortar and immersion time. The filler effect of RS on the ASR expansion of mortar was determined by Equation (2) when the immersion times of all mortars were the same.

(2) $$F_{Filler}=E_{RS}-E_{CT}$$

where *F_Filler_* is the filler effect of RS on the ASR expansion of the mortar (%), *E_RS_* is the ASR expansion of the RS-containing mortars (%), and *E_CT_* is the ASR expansion of the CT mortar (%).

The filler effect of finer particles improves the mechanical and durability properties of mortar or concrete. Since 33RS and 5RS were ground river sands and consisted mainly of quartz, which does not react with OPC hydration products, the different reductions of expansion between 33RS and 5RS mortars were considered to be predominantly due to the filler effect of the RS material.

The filler effect due to RS was an important factor in lowering the ASR expansion, and the filler effect had a higher degree of effectiveness at a later age. For instance, 5RS exhibited changes in the ASR mortar expansions of −0.177 and −0.283% at ages of 14 and 28 days, respectively, due to the filler effect. It should be noted that the negative values of the expansion indicate that there was a reduction of the ASR mortar expansion compared to the CT mortar. The reduction of the OPC content from 100 to 80% by weight meant there was a lower amount of Ca(OH)_2_ available to react with SiO_2_ in the reactive aggregate, and there was thus a lower content of ASR products. Moreover, Ca(OH)_2_ continually reacted with SiO_2_ in the reactive aggregate, and the filler effect could be observed clearly after 5 days of immersion, with more apparent results after longer immersion durations.

When the OPC comprised only 80%, the ASR expansion of the mortars incorporating 5RS and 33RS (with equal OPC contents in the mortar) was 0.177 and 0.122%, respectively, which were lower than the ASR expansion of the CT mortar. Meanwhile, increasing the fineness of RS, from 33RS to 5RS, produced an absolute increase in the ASR expansion of about 0.055% at 14 days, and 0.092% at 28 days. It is noted that the filler effect of RS (33RS and 5RS) was less effective than the 20% reduction of OPC in the binder, since the lowering of OPC in the mortar by 20% reduced the ASR expansion to about 0.122% (when using 33RS as a replacement for OPC) or 0.177% (when using 5RS as a replacement for OPC), while the reduction of the ASR expansion by the filler effect of 33RS and 5RS was only 0.055% at 14 days.

### 4.5. Effectiveness of FA in Controlling ASR Expansion of Mortar

The effectiveness of FA in controlling expansion due to ASR depended on the amount of FA used to substitute OPC. It was observed in previous research [31] that the expansion due to ASR was reduced by 27 to 87% when ground FA was used to replace OPC at 10 to 40%. The 5FA and 33FA mortars in this study reduced the ASR expansion by 57 and 52% at 14 days, respectively, suggesting that the different particle sizes of FA had a marginal effect on its ability to reduce the ASR expansion. Similarly, the replacement of 20% of the OPC with Class C FA in previous research showed reductions in ASR expansion in the range of 17 to 65% [40,41]. Meanwhile, the replacement of 30 to 40% of the OPC with Class C FA could reduce the ASR expansion in the range of 27 to 96% [29,40,41].

The use of 5RS and 33RS to replace 20% of the OPC reduced the ASR expansion of mortars at 14 days by 76 and 52%, respectively. The use of 33FA and 33RS provided similar reductions in the ASR expansion of the mortar, while the use of 5RS was more effective than the use of 5FA for the reduction of the ASR expansion. However, the replacement of OPC with RS resulted in a lower compressive strength of the mortars, compared to those with FA. Mortars containing 20% of 5RS and 33RS had a strength activity index of 89% and 78%, respectively, at the age of 28 days. In contrast, the mortars containing 20% of 5FA and 33FA had a strength activity index of 108 and 93%, respectively, at the age of 28 days [37].

## 5. Conclusions

Based on the results from this study of the influence of cement replacement with fly ash and ground sand with different fineness on the alkali-silica reaction of mortar, the conclusions can be summarized as follows:

1. The use of FA with 5 and 33 wt.% of its particles retained on a No. 325 sieve to replace 20% of OPC reduced the ASR expansion of the mortar. The extent of the reduction depended on the replacement of OPC by FA rather than on the fineness of FA.
2. The use of RS to partially substitute OPC reduced ASR expansion of the mortar by decreasing the Ca(OH)_2_ content available from OPC.
3. The filler effect of RS is a factor that contributed to the reduced ASR expansion of the mortar. Moreover, the filler effect of RS with a higher fineness was more effective than that of RS with a lower fineness.
4. The use of Class C FA with different levels of fineness (retained on No. 325 sieve at 5 and 33% by weight) to replace OPC at 20% reduced the ASR expansion by more than 50% at 14 days. In addition, it is suggested that reducing the CaO content (by reducing the OPC content) in mortar could be more effective in mitigating the ASR expansion than the filler effect of the cement replacement material.

## Figures and Tables

**Table 1:** Chemical compositions of ordinary Portland cement (OPC), 5FA, and 5RS.

Sample	Chemical Composition (%)									
	SiO_2_	Al_2_O_3_	Fe_2_O_3_	CaO	SO_3_	MgO	Na_2_O	K_2_O	Na_2_O_eq_	LOI
OPC	20.8	4.7	3.3	65.4	2.7	1.3	0.3	0.4	0.6	1.0
5FA	33.2	17.1	12.8	25.2	3.2	1.9	1.2	2.5	2.8	2.9
5RS	92.0	5.0	0.6	0.2	0.2	0.0	0.4	0.8	0.9	0.1

**Table 2:** Physical properties of materials.

Sample	Specific Gravity	Retained on a No. 325 Sieve (%)	Strength Activity Index (%)	
			7 days	28 days
OPC	3.14	20.0	—	—
5FA	2.41	5.0	86	105
33FA	2.33	32.8	81	93
5RS	2.72	4.8	67	68
33RS	2.63	32.5	62	62

**Table 3:** Mix proportions of mortar bars in the alkali–silica reaction (ASR) investigation.

Mortar Bars	Mix Proportions (By Weight)					
	Cement	FA	RS	Fine Aggregate	W/B	Flow (%)
CT	1.00	—	—	2.25	0.47	100
5FA	0.80	0.20	—	2.25	0.47	101
33FA	0.80	0.20	—	2.25	0.47	99
5RS	0.80	—	0.20	2.25	0.47	92
33RS	0.80	—	0.20	2.25	0.47	88

## Data Availability

The data presented in this study are available on request from the corresponding author.
